# A validated mouse model capable of recapitulating the protective effects of female sex hormones on ascending aortic aneurysms and dissections (AADs)

**DOI:** 10.14814/phy2.14631

**Published:** 2020-11-26

**Authors:** Xiaoyan Qi, Fen Wang, Changzoon Chun, Lennon Saldarriaga, Zhisheng Jiang, Eric Y. Pruitt, George J. Arnaoutakis, Gilbert R. Upchurch, Zhihua Jiang

**Affiliations:** ^1^ Division of Vascular Surgery and Endovascular Therapy University of Florida College of Medicine Gainesville FL USA; ^2^ Institute of Cardiovascular Disease University of South China Hengyang China; ^3^ Division of Thoracic and Cardiovascular Surgery University of Florida College of Medicine Gainesville FL USA

**Keywords:** aortic dissection, inflammation, model, rupture, sex

## Abstract

Fewer females develop AADs (ascending aortic aneurysms and dissections) and the reasons for this protection remain poorly understood. The present study seeks to develop a mouse model that may be utilized to address this sexual dimorphism. Adult normolipidemic mice were challenged with BAPN (β‐aminopropionitrile), AngII (angiotensin II), or BAPN + AngII. An initial protocol optimization found that 0.2% BAPN in drinking water plus AngII‐infusion at 1,000 ng kg^−1^ min^−1^ produced favorable rates of AAD rupture (~50%) and dilation (~40%) in 28 days. Using these dosages, further experiments revealed that BAPN is toxic to naïve mature aortas and it acted synergistically with AngII to promote aortic tears and dissections. BAPN + AngII provoked early infiltration of myeloid cells and subsequent recruitment of lymphoid cells to the aortic wall. AADs established with BAPN + AngII, but not AngII alone, continued to expand after the cessation of AngII‐infusion. This indefinite growth precipitated a 61% increase in the AAD diameter in 56 days. More importantly, with the optimized protocol, significant differences in AAD dilation (*p* = .012) and medial degeneration (*p* = .036) were detected between male and female mice. Treatment of ovariectomized mice with estradiol protected AAD formation (*p* = .014). In summary, this study developed a powerful mouse AAD model that can be used to study the sexual dimorphism in AAD formation.

## Graphical abstract

The aortic pathology in this murine AAD model features an early onset of intimal/medial tears followed by aortic rupture or progressive medial degeneration at the late stage. Chronic inflammation, possibly driven by cell death, substantiates AAD dilation in the absence of AngII infusion. Female sex hormones, particularly estrogen, protect against AAD formation in this model.

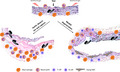

**New & Noteworthy**

This study created a novel mouse model and validated its application in studying the formation and sexually dimorphic presentation of human sporadic AADs.

## 1 INTRODUCTION

Ascending aortic aneurysm and dissection (AAD) is a life‐threatening disease with no effective medical therapies available to prevent or slow its development. The number of reported AADs increased by nearly 300% in the late 20th century (Ramanath et al., 2009) and is projected to escalate further in coming years (Theruvath et al., 2012). Current guidelines for clinical management recommend imaging surveillance of ascending aortic dilation (Ho et al., 2017). However, using this protocol, about 50% of patients will die before reaching a hospital (Golledge & Eagle, 2008) and a further 20% will die within 10 years (Golledge & Eagle, 2008), as dilated aortas are prone to dissect and rupture. Furthermore, dissection of small aneurysms is common in patients. It has been shown that the risk of aortic dissection does not correlate linearly with the degree of aortic dilation. Rather, it stays unchanged until the dilation approaches to 6.0 cm or greater in diameter (Evangelista et al., 2018; Goldfinger et al., 2014; Pape et al., 2007; Trimarchi et al., 2011). These clinical observations have led to a hypothesis that aortic dissection and aneurysms precede through distinct biological pathways (Golledge & Eagle, 2008). Nevertheless, teasing out these pathways has been challenging due to the silent nature of AADs and the lack of animal models capable of reliably recapitulating AAD development.

AADs may result from single‐gene mutations, such as those present in patients with Marfan syndrome, Loeys–Dietz syndrome, or familial thoracic aortic aneurysm and dissection (Lindsay & Dietz, 2011; Milewicz, et al., 2017). Because of these well‐defined genetic defects, mouse models carrying the pathogenic human alleles have been the most appropriate tools for studies to understand the dynamic pathogenic processes and screen drugs to treat these genetic aortic diseases (Gould et al., 2019; Milewicz, et al., 2017; Milewicz, et al., 2017). Mechanistic details, such as abnormal mechanosensing (Humphrey et al., 2014) and activation of the kinase network (Gallo et al., 2014; Gould et al., 2019; Lindsay & Dietz, 2011) have been implicated to drive medial degeneration and aortic dilation. However, aneurysms in these models begin to form at the developmental stage and rarely dissect or rupture. These features preclude the use of these models to study sporadic aortic dissections, a particular form of the disease that often penetrates normal aortas of subjects older than >60 years (LeMaire & Russell, 2011). To satisfy this unmet need, non‐genetic approaches, particularly insults of the aorta with chemicals, such as angiotensin II (AngII; Rateri et al., 2014), calcium chloride (Ikonomidis et al., 2003), and elastase (Pope et al., 2015), have been developed to induce aortic aneurysm formation. Success has also been achieved through post‐natal targeting of pathways essential for the maintenance of the aortic wall homeostasis, such as the mineralocorticoid and TGFβ pathways. This has been demonstrated by our laboratory (Schmit et al., (2015); Yang et al., 2016; Zhou et al., 2019) and others (Li et al., 2014; Liu et al., 2013). However, aneurysms in these models either do not dissect or rupture (Ikonomidis et al., 2003; Pope et al., 2015), or do so at an extremely low rate (<20%; Li et al., 2014; Rateri et al., 2014; Yang et al., 2016). A few studies (Fashandi et al., 2018; Izawa‐Ishizawa et al., 2019; Kanematsu et al., 2010; Kurihara et al., 2012) attempted to create a better model by modifying the traditional AngII protocol with the addition of β‐aminopropionitrile (BAPN). The modified protocols either boosted the rupture to an extremely high rate (>90%; Fashandi et al., 2018; Kurihara et al., 2012) or only marginally enhanced the rate of rupture (Hirakata et al., 2020; Izawa‐Ishizawa et al., 2019; Kanematsu et al., 2010). Additionally, none of these studies determined whether this modified version is capable of recapitulating sex‐dependent differences in AAD formation, as in the parent AngII model (Alsiraj et al., 2018).

In the current study, a mouse model was created and validated for its application in studying sporadic AADs. This model is uniquely characterized by early onset (in 3 days) of intimal/medial tears, frequent AAD rupture (50% in 4 weeks), and indefinite AAD dilation (>60% in 8 weeks). Additionally, the present model recapitulates sex‐dependent differences in AAD formation—a phenomenon termed “sexual dimorphism”—which was recently identified in patients with type A dissections (Jondeau et al., 2016; Pape et al., 2015). Furthermore, estrogen inhibited AAD formation in this model. To our knowledge, this is the first model that has been empirically validated for use to study the protective effects of female sex on AAD development.

## 2 METHODS

### 2.1 Animals

This study conforms to the Guide for the Care and Use of Laboratory Animals of the National Institutes of Health. The Institutional Animal Care and Use Committee of the University of Florida approved all procedures. Mice used in this study were wild type (WT) on a background of C57BL/6J, purchased from Jackson laboratory, and allowed to acclimate for a week prior to enrollment in the study.

### 2.2 Model creation

Beta‐aminopropionitrile (BAPN, A3134, Sigma‐Aldrich), a lysyl oxidase inhibitor, was delivered via drinking water at a concentration of 0.2% with the water refreshed twice weekly. Angiotensin II (AngII, 4006473, Bachem) was dissolved in saline and delivered via an osmotic pump (Alzet 2004; Daugherty et al., 2000; Schmit et al., 2015). The pumps were implanted subcutaneously 3 days after BAPN administration. Mice in control groups received pumps loaded with saline. All animals were checked daily for alertness and activeness to identify cases with aortic ruptures.

### 2.3 Necropsy

All animal corpora were necropsied to examine for aortic ruptures. Because of the technical difficulties in locating the actual break in the aortic wall, location of the nearby blood clot was taken as the marker for the site of aortic rupture. For example, cases with thrombi around the ascending segment, aortic arch, or in the pericardial cavity were deemed a “rupture of the ascending aorta.” When thrombi were present, but not stuck around a specific aortic region, the case was deemed a “rupture of the thoracic aorta.”

### 2.4 Ultrasound imaging

Animals were anesthetized via inhalation of 1.5%–2.0% isoflurane and placed in a supine position on a heated platform, followed by removing the fur in the area of imaging with a depilatory cream and warm water. A high‐resolution Vevo 2100 Imaging System with an MS550D (central frequency: 40 MHz) linear array transducer (VisualSonics) was then utilized to scan aortas under B‐mode. Ascending thoracic aortas (ATAs) were imaged via the right parasternal long‐axis view, with the transducer finely adjusted to a position that captures the largest possible width of the segment. A cine‐loop was then recorded and reviewed to select the frame that displays the widest ATA. On the selected image, lumen diameter of the ATA was measured using the straight‐line tool, with one end localized to the point of 11 o'clock of the right pulmonary artery and the other end landing to such a point of the opposite wall that the line symmetrically divides the trunk of the ATA (Yang et al., 2016; Zhou et al., 2019).

### 2.5 Ovariectomy and estrogen replacement

Ovariectomy (ovx) was performed on mice at an age of 6‐weeks via a dorsal midline skin incision caudal to the posterior boarder of the ribs. Once the ovary and the oviduct were exposed, a hemostat was used to crush the oviduct and the cranial‐most part of the uterus. Then the ovary was removed with cautery and the remaining tissue replaced into the peritoneal cavity. After both ovaries were removed, the skin incision was closed with metal clips. All OVXed mice were given a 3‐week interval to ensure the diminishment of the systemic, physiological level E2 effect before enrolling in experiments.

Homemade silastic capsules were utilized for in vivo delivery of 17β‐estradiol (E2, E8857, Sigma‐Aldrich). E2 was frequently administered to rodents through subcutaneous implantation of drug pellets. However, a study reported that the most frequently used E2 pellets produced an early peak of serum E2 concentration that is hundreds of times higher than the physiological level. To resolve this issue, the same group fabricated silastic capsules for E2 delivery and demonstrated that this method produces physiological levels of estrogen at a consistent rate in mice (Ingberg et al., 2012). Technical details are provided in a published video (Ström et al., 2012). This method has been successfully adopted by multiple groups (Ingberg et al., 2012; Thatcher et al., 2015). Briefly, segments (1.575 mm of inner diameter and 2.0 cm in length) of autoclaved silastic tubing (1118915G, Thermo Fisher Scientific) and wooden plugs (2.0 mm inner diameter and 3.0 mm in length, 22‐363‐156, Thermo Fisher Scientific) were made using a sterile blade. E2 was dissolved in autoclaved sesame oil (S3547, Sigma‐Aldrich) at a concentration of 36.0 µg/ml. Then, the silastic tubing segments were filled with the E2 solution or sesame oil and capped with the wooden plugs. After removing air bubbles, the capsules were incubated at 37°C overnight in a solution the same as the one loaded in the capsules. The OVXed mice were randomly assigned to two groups and received E2 or oil only capsules via subcutaneous implantation. A week after capsule implantation, mini pumps (Alzet 2004) were implanted in these mice to deliver AngII at a dosage equivalently administered to gonad‐intact male and female mice.

### 2.6 Gross evaluation

Aortas were evaluated in situ under an operating scope. Gross pathologies recorded during evaluation are as follows: periaortic adhesions (connective tissues adherent to the aortic wall), intramural hematoma (blood clot or yellow precipitates in the aortic wall), and aneurysm formation (focal dilation with an abrupt transition to the adjacent segments). Anatomic location of the pathologies was visually determined and assigned to one of the following regions: ascending/arch aorta (from the aortic root to the ligamentum arteriosum), descending thoracic aorta (from the left subclavian artery to the aortic hiatus), and abdominal aorta (the segment below the diaphragm).

### 2.7 Evans blue staining

Evans blue solution (5%) was prepared in saline and administered to mice through tail vein injection 30 min before tissue collection. During tissue collection, 5.0 ml of saline was injected through the left ventricle and drained through the right atrial appendage to thoroughly remove Evans blue in the blood, followed by perfusion fixation with 1.0 ml of 10% neutral buffered formalin. The ATAs were cut open longitudinally and spread on slides with the luminal side facing up. After mounting with coverslips, the specimens were evaluated microscopically for Evans blue extravasation and the presence of intimal/medial tears. For each specimen, the area with positive Evans blue staining was quantified using Zen lite 2012 (Carl Zeiss) and normalized to the area of luminal surface of the specimen. After completion of the *en face* evaluation, all specimens were paraffin‐embedded and sectioned to collect serial cross‐sections at 100.0 µm intervals. All sections were evaluated under bright field and fluorescence microscopy for Evans blue extravasation.

### 2.8 Histology

Cross‐sections (5.0 µm) were collected at locations 0, 100, and 200 µm from the proximal end of the AADs. The point where the first complete circle was obtained during sectioning was taken as “0 µm.” A set of cross‐sections were stained using Movat's staining protocol as described previously (Jiang et al., 2004, 2009) and evaluated by two blinded observers with expertise in the histology of murine AADs. A 5‐point score system, as we have described previously (Yang et al., 2016), was applied to access the severity of intimal/medial tears, intramural hematoma, and medial thinning/diminishment. For each AAD, the scores assigned to those pathologies at each location were summed, and the averageamongst the three locations was calculated to represent the degree of aneurysmal degeneration.

Morphometric measurements were performed on cross‐sections using a digital imaging system (Zen Lite 2012, Zeiss). The adventitia of AADs was defined as the layer with the dense matrix deposition. For each AAD sample, the area of the adventitia was normalized to the circumference of the external elastic lamina. This normalized value was taken as “thickness” of the adventitia.

### 2.9 Prussian blue staining

Prussian blue staining was performed with a kit purchased from Abcam (ab150674, Abcam). Briefly, paraffin sections were rehydrated, followed by incubation with a mixture containing 10% potassium ferrocyanide and 20% hydrochloric acid. After rinsing in water to remove the unreacted reagents, specimens were counterstained with nuclear fast red.

### 2.10 Immunohistochemistry and immunofluorescence staining

All assays were performed on formalin‐fixed, paraffin‐embedded sections. Antigens were unmasked by incubating specimens in citra buffer (pH 6.0, H3300, Vector Laboratories) heated and pressurized with a pressure cooker. After blocking non‐specific bindings, specimens were incubated with primary antibodies at 4°C overnight, followed by labeling with Alexa Fluor‐conjugated secondary antibodies at room temperature for 2 hr. Nuclei were counterstained with DAPI (D9542, Sigma‐Aldrich, St. Louis, MO, USA). Information for all antibodies is provided in Table 1.

**TABLE 1:** Information of antibodies used in this study

Antigen	Antibody	Vender	Titer
CD19	Rat anti‐mouse IgG2a	14019480,Thermo Fisher, Waltham, MA, USA	1:50
Ly6b.2	Rat anti‐mouse IgG2a	MCA771G, Bio‐Rad, Hercules, CA, USA	1:100
CD3	Rabbit anti‐mouse IgG	ab5690, Abcam, Waltham, MA, USA	1:200
CD68	Rabbit anti‐mouse IgG	PA5−78996, Thermo Fisher, Waltham, MA, USA	1:250
α‐actin	Cy3‐mouse IgG	C6198, Sigma‐Aldrich, St. Louis, MO, USA	1:400
Rat IgG	Alexa Fluor 546 goat IgG	A11081, Thermo Fisher, Waltham, MA, USA	1:200
Rat IgG	Alexa Fluor 488 goat IgG	A11006, Thermo Fisher, Waltham, MA, USA	1:200
Rabbit IgG	Alexa Fluor 488 goat IgG	A11008, Thermo Fisher, Waltham, MA, USA	1:200
Control	Normal rabbit IgG	NBP2‐ 24891, Novus Biologicals, Centennial, CO, USA	1:100
Control	Rat isotype IgG2a	02–9688, Thermo Fisher, Waltham, MA, USA	1:100

### 2.11 Statistical analysis

All data are expressed as the mean ± *SEM*. Statistical analyses were performed using Sigma Plot 14.0. Datasets were evaluated for normality and equivalence of variance. For those failing this evaluation, logarithmic and exponential transformations were performed to meet these requirements. Student's *t*‐test, two‐way ANOVA, and two‐way repeated‐measures ANOVA, weighted log rank survival test, and Mann–Whitney Rank Sum test were performed, when appropriate, with Holm–Sidak analysis being used for post hoc tests. *p* < .05 was considered statistically significant.

## 3 RESULTS

**FIGURE 1:**
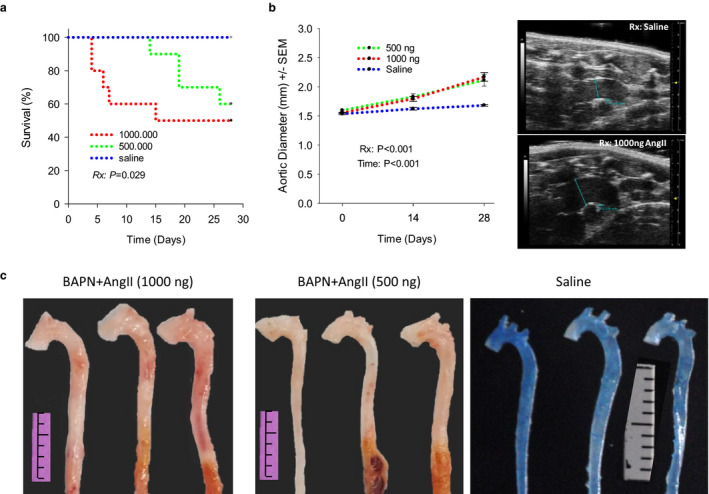
The combined challenge of BAPN and AngII induces acute aortic rupture and AAD formation in adult normolipidemic mice. (a) Weighted log rank survival analysis. Reduction of AngII from 1,000 ng kg^−1^ min^−1^ (*n* = 10) to 500 ng kg^−1^ min^−1^ (*n* = 10) postponed aortic rupture (*p* = .029). (b) Dilation of the AADs evaluated with ultrasound imaging. Reduction of AngII dosage to 500 ng did not slow down aortic dilation (*p* = .897, two‐way repeated‐measures ANOVA). (c) Gross specimens collected on d28. Segments below the level of the diaphragm were cropped off. Blue color in the saline control group resulted from the Evans blue administered prior to gross evaluation. Ruler scale: 1.0 mm. Note the similar enlargement of the ascending aorta and the aortic arch between groups subjected to low and high AngII dosages

*Challenge with BAPN and AngII in concert promotes aortic rupture and perpetuates progressive AAD development in adult, normolipidemic mice*. Recently, we demonstrated the use of BAPN to overcome the growth‐resistance of AAA formation induced by elastase (Lu et al., 2017) and to boost aortic rupture in AngII‐infused hyperlipidemic mice (Fashandi et al., 2018). The current experiment evaluated the efficacy of the same BAPN dosing regimen on AAD formation induced by AngII‐infusion in normolipidemic mice. BAPN (0.2%) was administered to adult (10–15 weeks of age) C57BL/6J male mice in their drinking water. Three days later, AngII was infused into these mice at a rate of 1,000 ng kg^−1^ min^−1^ (*n* = 10) or 500 ng kg^−1^ min^−1^ (*n* = 10). Mice in the control group (*n* = 6) received mini pumps loaded with saline and were on drinking water free of BAPN. A group of C57BL/6J female mice (10–15 weeks of age, *n* = 10) challenged with 0.2% BAPN and AngII (1,000 ng kg^−1^ min^−1^) were included in this experiment to decipher sex‐related differences in AAD formation. All animals were followed for 4 weeks. In male mice receiving the “standard” dose of AngII (1,000 ng kg^−1^ min^−1^), aortic rupture occurred as early as 4 days. Half of them (5 in 10) died from aortic rupture (3 AAD and 2 AAA ruptures) and all deaths were noted in the first 2 weeks. Reduction of AngII dosage significantly delayed (*p* = .029) the onset of aortic rupture, while had only modest impact on the rate of aortic rupture (3 AAD and 1 AAA ruptures, Figure 1a). As expected, aortic ruptures were located randomly in thoracic (6 of 9, Figure S1; https://figshare.com/s/781c619933cabd3759c3) and abdominal (3 of 9) aortas (*p* > .05). Ultrasound imaging revealed a progressive dilation of the AADs (*p* < .001). Reduction of the AngII‐dosage did not slow the aortic dilation. In 4 weeks, AADs forming under the high and the low dosage of AngII were dilated by 40% and 32%, respectively, while the control aortas only had a slight increase (<10%, *p* > .05) in diameter (Figure 1b). Gross examination detected peri‐aortic adhesions, intramural hematoma, and/or aneurysm formation in all ascending and frequently (11 in 12) in descending aortas (Figure 1c). Neither the prevalence nor the severity of these phenotypic traits was significantly different between groups challenged with high or low dosages of AngII (data not shown). All aortas of the control mice appeared normal (Figure 1c).

Similar to the success in AAD induction, AAAs (data not shown) were induced in these mice at a penetrance much greater than that previously documented for normolipidemic and hyperlipidemic mice (Rateri et al., 2014; Wang et al., 2010). While the high penetrance indicates that this model may hold advantages over the traditional AngII model, full characterization of AAA formation is necessary to determine the model performance (data not included) and its merit for AAA studies.

*BAPN is toxic to mature aortas and acts synergistically with AngII to promote AAD formation*. One of the limitations of the experimental aneurysms induced with chemicals is the lack of pathological complexity displayed by human aortic aneurysms. We have previously demonstrated that the addition of BAPN to the challenging regimen in adult mice improves the model performance (Fashandi et al., 2018; Lu et al., 2017). It was reported that BAPN is not toxic to mature aortas (Julian et al., 1979; Kumar et al., 1990), which implies that exacerbation of aneurysm formation results from its effect on tissue healing during progression, instead of initiation. We tested this hypothesis in the present study. Normolipidemic adult C57BL/6J male mice (10 weeks of age) were challenged with BAPN only (0.2%, *n* = 10, pumps loaded with saline), AngII only (1,000 ng kg^−1^ min^−1^, *n* = 10), or both BAPN and AngII (BAPN + AngII, *n* = 15). A subset of mice in each group (*n* = 3) were evaluated with Evans blue staining 3 days (d3) after AngII‐infusion. The remaining mice were followed up with ultrasound imaging for 56 days. Osmotic pumps in these mice were removed on d28 to determine whether the established AADs continue to expand in the absence of AngII.

**FIGURE 2:**
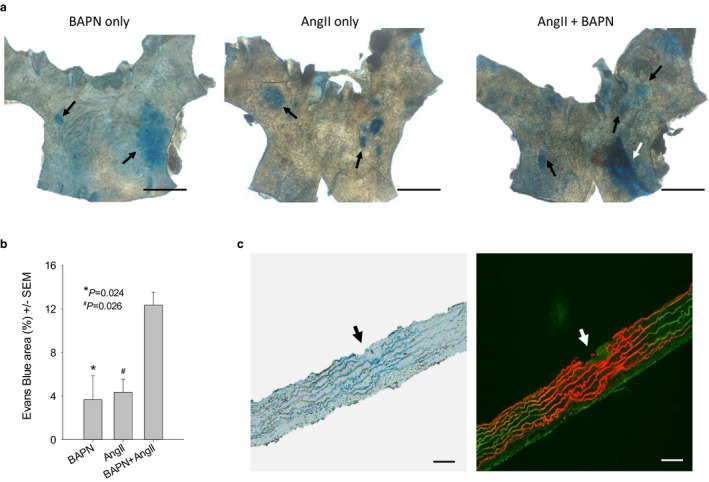
BAPN and AngII act synergistically to promote acute intimal/medial tears in the ascending aorta. Specimens were collected 3 days after AngII‐infusion. (a) En face view of the luminal surface of ATAs challenged with the indicated substances. Areas with extravasation of Evans Blue appeared in blue (black arrows). Note the extravasation of Evans Blue in the ATA challenged with BAPN only and a long intimal/medial tear (white arrow) in the ATA challenged with BAPN + AngII. Scaler bars: 1.0 mm. (b) Fractional area positive for Evans Blue staining (*n* = 3 per treatment). Data were analyzed using one‐way ANOVA. (c) Microscopic evaluation of cross‐sections of the ATAs. Left panel: bright field microscopy. An intimal/medial tear (black arrow) was located at the center of the area where elastic fibers were stained in blue. Right panel: fluorescence microscopy. Red: elastic fibers stained by Evans blue (Evans blue emits red fluorescence); green: autofluorescence. Note the co‐localization of Evans Blue extravasation with breaks of the intimal layer and elastic fibers (white arrow). Scale bars: 100 µm

Extravasation of Evans blue is an indicator of impairment of the barrier function of the endothelial monolayer. Unexpectedly, *en face* microscopy of the luminal surface detected isolated areas of positive Evan blue staining in all ATAs (ascending thoracic aortas, Figure 2a) treated with BAPN only. Some positive areas co‐localized with intimal/medial tears that were longitudinally oriented, while others were located in areas with physically intact luminal surface, indicating that functional damage precedes the structural destruction of the intimal layer. Clearly, BAPN is toxic to not only immature aortas but also mature aortas. ATAs of the AngII only and the BAPN + AngII groups all displayed abundant isolated areas of Evans blue extravasation and intimal/medial tears (Figure 2a). Morphometric analysis revealed that ATAs of the BAPN + AngII group had a significantly larger fraction of the Evan blue positive luminal surface than those of the BAPN only (Figure 2b, *p* = .024) and the AngII only (Figure 2b, *p* = .026) groups. Note that the mean of the BAPN + AngII group is much greater than the sum of the mean of BAPN only and AngII only groups (Figure 2b), indicating that BAPN acts synergistically with AngII to induce intimal/medial damage at the initiation stage.

Microscopic evaluation of serial sections of AADs collected on d3 revealed that Evans blue primarily stained the elastic laminae and made these sheets appearing in blue and red under bright field (Figure 2c, left panel) and fluorescence microscopy (Figure 2c, right panel; green: autofluorescence), respectively. Breaks of the intimal monolayer and the underlying elastin fibers were frequently detected and co‐localized to areas with positive Evans blue staining (Figure 2c). These findings demonstrate that the tearing of the intimal/medial layers occurs early in this model.

**FIGURE 3:**
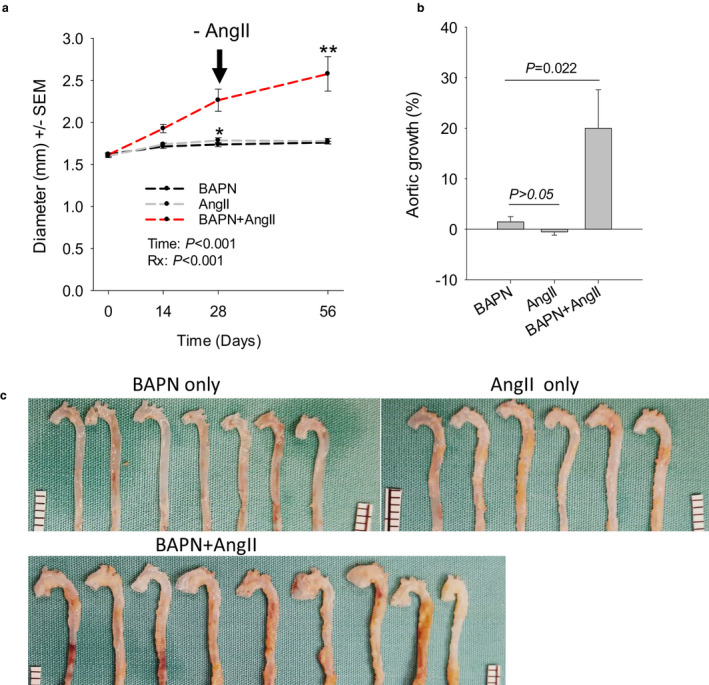
AADs induced by BAPN + AngII continue to expand in the absence of AngII. (a) Dilation of the AADs (*n* = 7–12) before and after discontinuation of AngII‐infusion (‐AngII). Osmotic pumps loaded with AngII or saline control were removed on d28 (black arrow). Note that only the AADs of the group initially challenged with both BAPN and AngII expanded persistently after cessation of AngII‐infusion. *p = .429, AngII versus BAPN; **p = .017, d56 versus d28 for the BAPN + AngII group. Data were analyzed using two‐way repeated‐measures ANOVA. (b) Dilation of the established AADs in the absence of AngII‐stimulation. Data were expressed as percent increase of the AAD diameter in the last 4 weeks and analyzed using one‐way ANOVA. (c) Gross specimens collected on d56. Aortic segments below the level of diaphragm were cropped off. Ruler scale: 1.0 mm. Note the severe dilation of ATAs and presence of intramural hematoma in the descending aorta of the BAPN + AngII group

*Prolonged AngII‐infusion is not required for established AADs to continue to expand in the BAPN + AngII model*. In the traditional AAA model induced by AngII, prolonged AngII‐infusion is required for established AAAs to continue to grow (Rateri et al., 2011). The present study examined the growth of established AADs in the absence of AngII. Discontinuation of AngII‐infusion was assured by removing the implanted osmotic pumps 4 weeks (d28) after implantation. Thereafter, animals were followed for another 4‐week period until d56. During the first 4‐week period, a slight, but significant, increase in lumen diameter was detected for AADs of the BAPN only (7%, *p* = .023) and AngII only (12%, *p* = .001) groups. During the following 4‐week period, AADs of both groups presented only modest changes in lumen diameter (Figure 3a). In contrast, a remarkable increase (41%) in lumen diameter was detected for AADs of the BAPN + AngII group at the end of the initial 4‐week period (Figure 3a). These AADs continued to dilate in the absence of AngII‐infusion, which resulted in a further 20% increase of lumen diameter (Figure 3a) in the second 4‐week period. In 8 weeks, AADs of this group gained a 61% increase in lumen diameter, which technically certifies these AADs as true aneurysms.

*The pathology of AADs induced by BAPN and AngII is characterized by progressive medial degeneration*. In order to define the temporal sequence of aneurysm degeneration, AADs from a group of mice (C57BL/6, male, 10 weeks of age, *n* = 10) challenged with BAPN + AngII were collected on d14, a mid‐stage time point that was missing from the aforementioned experiments. On gross examination, evident pathologies in thoracic aortas were limited to peri‐aortic adhesions and dotted intramural hemorrhage with no aneurysms noted in this cohort.

**FIGURE 4:**
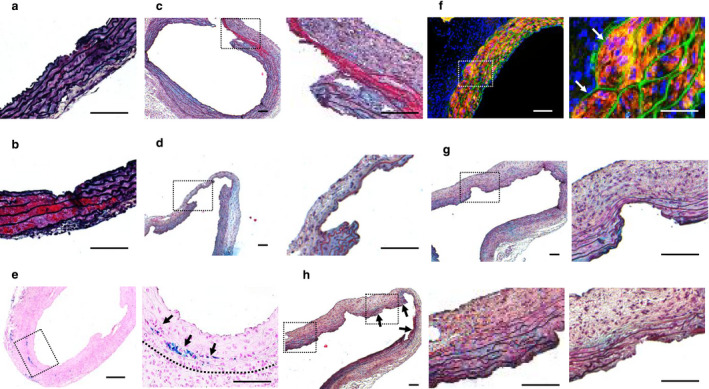
AADs induced by BAPN + AngII advance the aneurysmal degeneration through a sequence of pathologies. All specimens were stained with Movat's staining except those in E (Prussian blue staining) and F (immunofluorescence staining for α‐actin). (a and b) intimal/medial tears (a, arrows) and intramural hematoma (b, asterisks) presented by AADs at d3. (c and d) Full thickness intimal/medial tears (c) and penetrating aortic ulcers (PAUs) presented by AADs at d14. (e and f) Resolved hematoma (e, arrows) and widening of interlaminar space by hyperplastic SMCs (f) detected on d14 AADs. (g and h) PAUs (g) and medial diminishment (h, arrows) exhibited by d28 AADs. Areas with and without medial diminishment are shown with images placed in the mid and right, respectively, in the panel H. Scale bars: 100 µm

Histology of AADs was evaluated with Movat's staining. At d3, elastic fiber breaks and intimal/medial tears were readily detectable in all AADs. Some tears penetrated into the wall that otherwise looked structurally intact (Figure 4a), while others were connected to channels filled with blood (Figure 4b). By d14, several major pathologies that were infrequently detected at d3 became prominent. First, full‐thickness intimal/medial tears (defined as breaks of the intimal and medial layers with edges of the break being coarse and physically complementary) were frequently detected at multiple locations, with some serving as an entry point for blood to dissect the aortic wall (Figure 4c). Second, penetrating aortic ulcer (PAU, defined as an intimal/medial defect with an “erosion surface”) was detected on all AAD sections, varying considerably in number, size, and depth among individual AADs (Figure 4d). The third major pathology is resolved intramural hematoma. Prussian blue staining revealed absorbed hematoma in the interlaminar space in half of the examined AADs (Figure 4e), indicating that intramural hemorrhage is a frequent early event in this model. The fourth major pathology is the widening of the interlaminar space. Immunofluorescence staining showed that some interlaminar spaces were distended by cell‐crowds expressing smooth muscle α‐actin (Figure 4f), indicating that smooth muscle cells (SMCs) were activated to undergo hyperplastic response in the AADs. The fifth major pathology is medial thinning and diminishment. This pathology was detected occasionally at d28 (Figure 4h). However, the majority of AADs (6 in 9) presented with similar pathology at d56 (Figure S2). The last major pathology is the thickening of the tunic adventitia. This pathology was notable at d14 (Figure 4c) and remained evident at d28 (Figure 4g,h). By d56, it displayed dramatic in‐sample variation, with the actual thickness much thinner in areas with than in those without medial diminishment (Figure S2). These histologic findings suggest that the aortic wall undergoes a sequence of medial degeneration, perpetuating progressive dilation of the AADs.

**FIGURE 5:**
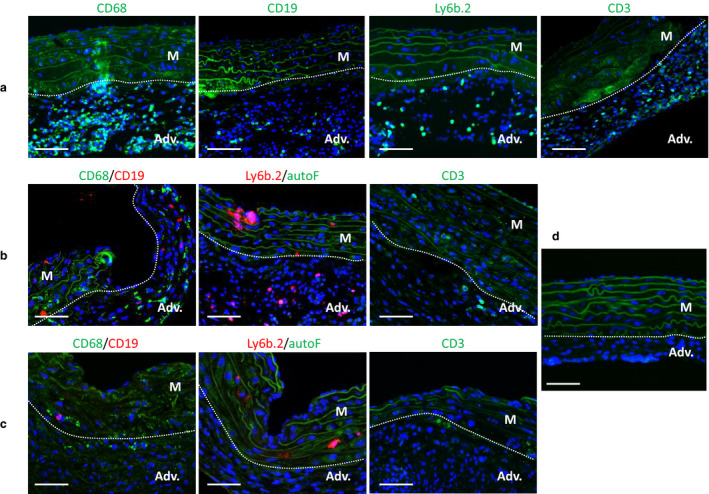
The challenge with BAPN + AngII induces the massive influx of macrophage, neutrophil, and B‐cell to AADs. (a) Immunofluorescence staining of markers specific for macrophage (CD68), B‐cell (CD19), neutrophil (Ly6b.2), and T‐cell (CD3). Specimens were collected 3 days after AngII‐fusion (d3). (b and c) Immunofluorescence labeling of the indicated inflammatory infiltrates in AADs collected on d14 (b) and d28 (c). (d) A representative image for negative controls in which the primary antibodies were replaced with normal rabbit IgG and rat isotype IgG2a. White dash lines depict the border between medial and adventitial layers. Adv., adventitia; AutoF, autofluorescence; M, media. Scale bars: 50 µm

*Aortic challenge with BAPN + AngII provokes acute inflammation that subsequently transitions to a chronic process*. AADs collected on d3 displayed an intense inflammatory response, characterized by the recruitment of massive numbers of macrophages as well as abundant neutrophils, B‐cells, and T‐cells. Interestingly, the vast majority of these cells were located in the adventitial layer (Figure 5a), indicating that adventitia is the “hot spot” of the inflammatory response at the early stage. At d14, inflammatory cells displayed a pattern of spatial distribution distinct from that detected at d3. In contrast to the diffuse distribution in the adventitial layer at d3, these cells clustered near structural defects, such as intimal/medial tears and PAUs, and penetrated across all layers of the aortic wall (Figure 5b). Additionally, a reduction in the number of major inflammatory subsets was microscopically evident in the adventitial layer of the AADs on d14 relative to d3 (Figure 5a,b), indicating the resolution of the inflammation. By d28, the inflammation was resolved to a greater degree, reflected by fewer inflammatory infiltrates, particularly macrophages, B‐cells, and T‐cells, in the AADs (Figure 5c). However, neutrophils were found clustering around intimal/medial tears (Figure 5c). It appears that, despite the bland nature of the initial inflammatory response in the aortic wall, acute inflammation can still “break out” in a restricted manner and cause more tissue damage in later stages of AAD development.

**FIGURE 6:**
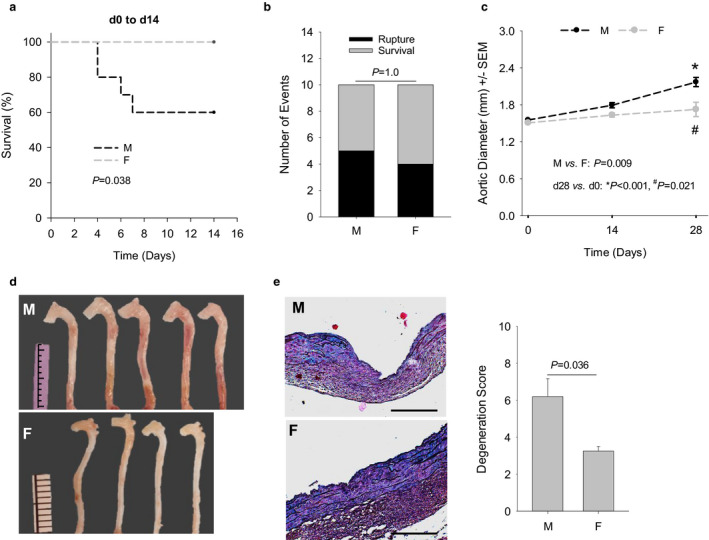
The BAPN + AngII model is capable of recapitulating the protective effect of female sex on type A aortic dissections. (a) Survival of mice in the first 2 weeks of AAD induction. M: male mice; F: female mice. (b) Number of mice either dead from aortic rupture or alive by d28. (c) Dilation of the AADs evaluated by ultrasound imaging. Data were analyzed using two‐way repeated‐measures ANOVA. (d) Gross specimens collected on d28. Note the difference in the width of the AADs between male and female mice. Ruler scale: 1.0 mm. (e) Medial degeneration evaluated with Movat's staining (*n* = 4–5). Representative images of male and female AADs are provided in the left panel, with the degeneration score assigned to these AADs plotted in the right panel. Data were analyzed using unpaired *t* test. Scare bars: 100 µm

*BAPN + AngII model recapitulates the protective effects of the female sex on AAD formation*. AADs differentially penetrate men and women, with the incidence in men nearly twice as much as that in women (Holmes et al., 2013; Olsson et al., 2006). We tested whether the BAPN + AngII model developed in the present study could recapitulate the dimorphic AAD formation between male and female mice. Adult (10–15 weeks of age) C57BL/6J male (*n* = 10) and female mice (*n* = 10) were treated with BAPN (0.2%) and AngII (1,000 ng kg^−1^ min^−1^) as described above and followed up with ultrasound imaging for 4 weeks. In the male cohort, five mice died from aortic rupture (2 AADs and 3 AAAs), with four ruptures occurring in the first week and none noted in the last 2 weeks. In contrast, ruptures did not occur in the female cohort in the first 2 weeks. Weighted log‐rank survival analysis showed a significant female advantage of survival in the first 2 weeks (Figure 6a, *p* = .038). However, four ruptures (1 AAD and 3 AAAs) occurred in female mice in the last 2 weeks. This “catch‐up” rendered the survival rate similar between male and female mice (Figure 6b).

Ultrasound imaging revealed that AADs of the female mice dilated to a significantly smaller degree than the male mice in 4 weeks (19% vs. 40%, *p* = .012, Figure 6c). On gross examination, female AADs presented similar major pathologies (namely peri‐aortic adhesions, intramural hematoma, and/or aneurysm formation) as those detected in male AADs (Figure 6d), and the prevalence of these pathologies was similar between AADs in both genders (data not shown). Medial degeneration, including intimal/medial tears, PAUs, and medial thinning, was microscopically evident in both male and female AADs. However, female AADs had lower scores for medial degeneration than male AADs (*p* = .036, Figure 5e), indicating that female mice preserved the aortic structure better than male mice.

**FIGURE 7:**
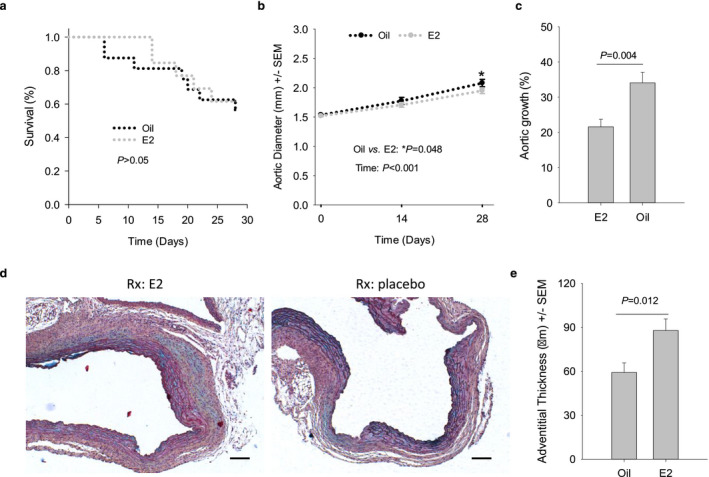
β‐estradiol (E2) attenuates the dilation of the AADs induced in OVXed female mice. (a) Survival of mice in 4 weeks. Note that aortic rupture in OVXed, E2‐treated mice did not occur until d14. (b) Dilation of the AADs estimated with ultrasound imaging. Data were analyzed using two‐way repeated‐measures ANOVA. (c) Changes in the AAD diameter over 4 weeks of AngII‐infusion. Data were analyzed using unpaired *t* test. (d) Representative images of AADs subjected to the indicated treatments. Scale bars: 100 µm. Note the difference in adventitial thickness between those AADs. (e) Adventitial thickening in the AADs treated with the indicated reagents (*n* = 8–9). Data were acquired analyzed using unpaired *t* test. Histologic data presented in E and F were obtained from AADs collected 28 days after AngII‐infusion

*Estrogen is a critical contributor to the protective effects of female sex on AAD formation*. Although several studies (Martin‐McNulty et al., 2003; Thatcher et al., 2015) including those from our group (Hannawa et al., 2009; Johnston et al., 2015) have demonstrated that female hormones, especially estrogen, are important players in mediating female‐protection against AAA formation, it is unclear whether estrogen plays a similar role in AAD formation. To address this issue, female mice with ovaries surgically removed at an age of 6 weeks were randomly assigned to receive 17‐β estradiol (E2, *n* = 13) or placebo (oil, *n* = 16) 3 weeks after ovariectomy (ovx). A week after E2 replacement, animals were infused with AngII (1,000 ng kg^−1^ min^−1^) and followed up for 28 days. In the placebo group, aortic ruptures were noted at both the early and the advanced stages (Figure 7a,b), resulting in a survival trajectory mirroring that presented by male mice (Figure 6a,b). E2 replacement resulted in a trend to delay the onset of aortic rupture at the early stage (Figure 7a), but failed to hold up as those with intact ovaries did in the first 2 weeks (Figure 6a). Thereafter, E2‐treated mice produced a survival trajectory similar to those treated with placebo (Figure 7b). It is worth noting that E2 replacement was unable to mitigate aortic rupture compared to the non‐OVXed female mice, nor did ovx promote aortic rupture compared to male mice (Figure S2a). These two factors might have contributed to the insignificant difference in survival between E2 and placebo groups. Ultrasound imaging revealed progressive dilation of AADs for mice treated with E2 or placebo (*p* < .01, Figure 7c). However, those treated with E2 displayed a much slower aortic dilation than those receiving placebo in 4 weeks (*p* = .004, Figure 7d).

In order to see how well E2 replacement restored the female advantage in preventing AAD formation, growth of the AADs was plotted for the groups of non‐ovx F (female with intact ovaries), M (male mice with intact gonads), ovx + E2, and ovx + oil in the same graph (Figure S2b). In contrast to the “E2‐refractory” aortic rupture, aortic growth in OVXed female mice responded to E2 with a rate similar to that experienced by gonad‐intact female counterparts (Figure S2b). Gross evaluation of aortas did not identify significant differences in the prevalence of the major aortic pathologies in AADs (Figure S3a) and other anatomic locations (data not shown) between the two groups. Compared with those receiving placebo, mice treated with E2 were protected against uteri involution resulting from ovx (Figure 3b), indicating that our homemade capsules had delivered an effective dosage of E2 to the OVXed mice.

Under microscopic evaluation, all AADs presented intimal/medial tears, medial thinning, and deposition of proteoglycans in the medial and the adventitial layers (Figure 7e). A few AADs developed penetrating ulcers and fresh intramural hematoma (data not shown). Medial degeneration scores for these AADs showed no significant difference between groups treated with E2 or placebo (data not shown). However, evident differences in adventitial thickening were noted during microscopic evaluation (Figure 7e). Subsequent morphometric analysis confirmed this observation, showing that E2‐treated AADs assembled a significantly thicker layer of adventitia than those treated with placebo (Figure 7f).

## 4 DISCUSSION

The present study created a powerful, new mouse AAD model and comprehensively characterized its capacity to recapitulate pathological features, including sexual dimorphism, of human sporadic AADs. This model is different in multiple ways from previously published models. First, AAD rupture occurs at a reasonable rate (50%). This rate is high enough to be taken as a meaningful endpoint for interventional and pharmacological studies, but not so high that it precludes opportunities to collect fresh tissue for mechanistic understanding of the underlying biology. Second, AAD dilates at an advanced rate, precipitating a 61% increase in the aortic diameter in 8 weeks. This dilation not only meets the threshold for the definition of an aneurysm, but also increases the level of AAD dilation far above natural aortic growth, which makes this model statistically powerful. Finally, AADs forming in this model exhibit sexually dimorphic phenotypes and are responsive to altered levels of female sex hormones. With these multiple characteristics similar to human AAD, this model will provide a useful tool for studies aiming to address critical issues related to sporadic AADs.

AngII is commonly infused into hyperlipidemic mice (Alsiraj et al., 2018; Daugherty et al., 2010; Wu et al., 2017), as previous experience in AAA creation warns that this comorbidity might be indispensable for AAD‐induction (Senemaud et al., 2017). Unlike AAAs that are frequently coupled with atherosclerosis (Nordon et al., 2011; Toghill et al., 2017), AADs are rarely associated with atherosclerosis (LeMaire & Russell, 2011). Therefore, hyperlipidemia might be irrelevant and a confounding factor in that AAD model. An elegant study addressed this issue and showed that the elimination of hyperlipidemia does not affect the incidence and severity of AAD formation in AngII‐infused mice (Rateri et al., 2014). However, this refinement was unable to triumph over the concern of low AAD rupture rate (<10% [Rateri et al., 2014; Trachet et al., 2016]) and a moderate increase in AAD diameter (near 20% [Daugherty et al., 2010; Trachet et al., 2016; Wu et al., 2017]) in normolipidemic mice. The same drawback exists in the AAD model created with BAPN. BAPN is an aortic toxin postulated to affect only immature aortas (Julian et al., 1979; Mc, 1958). Accordingly, the common practice is to deliver this compound to mice at a weaning age (Kurihara et al., 2012; Ren et al., 2016), which brings into question its application to study sporadic AADs, a disease with an onset age >60 years (LeMaire & Russell, 2011). A few studies attempted to address these issues by challenging adult, normolipidemic mice with both BAPN and AngII. These studies successfully improved the incidence of AAD formation to greater than 80% (Hirakata et al., 2020; Izawa‐Ishizawa et al., 2019; Kanematsu et al., 2010). However, they were unable to boost the dilation of the AADs, which is the most critical marker used in clinical surveillance. The rate of aortic rupture was also approximately 30%, which barely makes it a meaningful endpoint. In the current study, these issues were resolved by modifying the dosage and delivery of BAPN. This modification promoted the rate of aortic rupture to 50% in 4 weeks and resulted in AAD dilation by >60% in 8 weeks. We have previously created an AAD model through the deletion of smooth muscle Tgfbr1 (*Tgfbr1^iko^*; Liao et al., 2017; Yang et al., 2016; Zhou et al., 2019). Because of the distinct triggering mechanisms, these AAD models comprise a multi‐faceted platform for the cross‐model validation of scientific findings.

The BAPN + AngII model created in the current study has met the challenge in modeling the indefinite growth of human AADs. Rateri et al. (2011) reported that in the traditional AngII model, prolonged AngII‐infusion is required for an established aneurysm to continue to expand. In the present BAPN + AngII model, however, dilation of the AADs continued after cessation of AngII‐infusion. We have previously reported that BAPN breaks the growth‐resistance of mouse AAAs induced by elastase (Lu et al., 2017). Although not directly tested, it appears that the same mechanism applies for the growth of AADs in this model. The indefinite growth of AADs was associated with progressive medial thinning and diminishment. SMC depletion is a hallmark pathology of human aortic aneurysms, and in particular, thoracic aortic aneurysms and dissections (Halushka et al., 2016; Homme et al., 2006). This cellular event might have contributed to the medial diminishment observed for the advanced AADs. Early work in the field showed that the toxicity of BAPN is limited to immature aortas, with mature aortas being resistant to this vascular toxin due to their low rates of matrix turnover (Julian et al., 1979; Mc, 1958). Because of this traditional notion, it is generally assumed that in adult mice, BAPN facilitates the progression of aortic aneurysms via impairing the “wound‐healing” process. However, results obtained in the present study demonstrate that BAPN alone induces intimal/medial tears in mature ascending aortas. This indicates that BAPN is engaged in both the initiation and the progression phases during AAD development.

Murine AAD models created with BAPN or AngII are recognized for their capacity of mimicking the inflammatory aspect of human AADs (Daugherty et al., 2010; Rateri et al., 2014; Ren et al., 2018; Wu et al., 2017). The present BAPN + AngII model has inherited this capacity from its parental models, evidenced by the massive recruitment of macrophages, neutrophils, and lymphocytes that begins in the adventitia of AADs. Inflammation has been identified as one of the critical mechanisms responsible for aneurysm formation in mice challenged with BAPN (Ren et al., 2018) or AngII (Daugherty et al., 2010; Wu et al., 2017). This indicates that the same mechanism applies to AAD formation in the present BAPN + AngII model.

Owens et al reported that AngII stimulates the hyperplastic response of medial SMCs in the mouse ascending aorta (Owens et al., 2010). Phenotypic switching is a well‐recognized mechanism through which SMCs contribute to aortic aneurysm formation (Alexander & Owens, 2012; Petsophonsakul et al., 2019). In echoing this notion, we found that SMCs piled up in the interlaminar space of the AADs. However, further studies are needed to determine the clinical relevance of this cellular event since human AADs generally exhibit SMC depletion rather than proliferation (Milewicz, et al., 2017).

Gender is an established risk factor for both thoracic and abdominal aortic aneurysms. While multiple animal models have been developed for recapitulating sexual dimorphism in AAA formation (Alsiraj et al., 2017; Hannawa et al., 2009), models validated for use in studying sexual dimorphism in AAD formation are scarce (Alsiraj et al., 2018). The present BAPN + AngII model displayed differential dilation of AADs between male and female mice and responded to the manipulation of female sex hormones with measurable phenotypic differences. These results have validated the use of this model in studying sexual dimorphism in AAD formation. We have previously demonstrated a critical role for estrogen in protecting against AAA formation (Hannawa et al., 2009; Johnston et al., 2015). The present study extended this concept to AAD formation.

Our results showed that ovariectomy could not fully reprogram female AADs to grow like male AADs, and E2 replacement was unable to fully rescue AAD dilation and rupture in OVXed female mice. These results are in line with the concept that in addition to estrogen, other components, such as progesterone, sex chromosomes (Alsiraj et al., 2018), and male hormones (Zhang et al., 2012), are engaged in dictating differential phenotypic expression between genders. Additionally, the results underscore some of the limitations of the murine ovariectomy model. For example, we have previously shown that in OVXed mice, the residual estrogen secreted by peripheral organs and fat can significantly protect AAA formation although to a lesser degree than the physiological levels of estrogen released by peripheral and central sources (Johnston et al., 2015). Furthermore, E2 replacement restores only one of the many hormones produced in ovaries (Broekmans et al., 2009).

In summary, a novel mouse AAD model was created featuring an appropriate rate of aortic rupture with continued aortic dilation. Using this model, we demonstrated for the first time that estrogen protects against AAD formation. This AAD model will allow investigators to pursue answers to key questions in the hopes of better understanding the pathogenesis, including gender differences, of sporadic human AADs. Preclinical studies may also be performed with this model to develop targeted medical therapies for the treatment of sporadic human AADs.

## AUTHOR CONTRIBUTIONS

X Qi, Z Jiang^2^, G. R. Upchurch Jr., and Z. Jiang^1^ designed the research; X Qi, F. Wang, X. C Chun, L Saldarriaga, E.Y. Pruitt, and G.J. Arnaoutakis performed the research; X Qi and Z Jiang^1^ analyzed the data; X Qi, Z Jiang^2^, G. R. Upchurch Jr., and Z. Jiang^1^ wrote the paper.

## Supporting information



Supplementary Material
