# Supplementary material for: A validated mouse model capable of recapitulating the protective effects of female sex hormones on ascending aortic aneurysms and dissections (AADs)
Source: Physiol Rep. 2020 Nov 26;8(22):e14631. doi: 10.14814/phy2.14631 (PMC7690909; doi:10.14814/phy2.14631)
Supplement: Supplementary file 1 — Supplementary Material [file PHY2-8-e14631-s001.docx]

**A validated mouse model capable of recapitulating the protective effects of female sex hormones on ascending aortic aneurysms and dissections (AADs)**

Xiaoyan Qi^1,2^, Fen Wang^1^, Changzoon Chun^1^, Lennon Saldarriaga^1^, Zhisheng Jiang^2,*^, Eric Pruitt^1^, George Arnaoutakisi^1, 3^, Gilbert R. Upchurch Jr^1^, Zhihua Jiang^1,*^

**Supplemental Data**

Figure S1. Grass image of a ruptured AAD. This mouse died five days after AngII-infusion. It had a hemothorax in the left chest. A big size of blood clot was located around the ascending and the arch area. After removing the clot, an opening hole was noted in the anterior wall of the ascending aorta.

Figure S2. The indefinite AAD growth is associated with severe medial degeneration. Mice were initially challenged with the indicated reagents for four weeks and followed up for another four weeks after the implanted pumps were removed. Administration of BAPN to the groups that were initially on BAPN continued during the extended follow-up period. Representative Movat’s staining images were provided for each group. Upper panel, Cross section of AADs. Note the difference in size of the aortic rings and integrity of the wall structure among AADs induced by different reagents. Lower panel, Higher power view of the boxed region of images shown in the upper panel. Note the difference in architectures of the medial layer among the AADs. Areas with medial diminishment were randomly distributed in AADs induced by BANP+AngII, but not detected in those induced by BAN or AngII.

Figure S3. Efficiency of ovx with and without E2 replacement on modeling sex-biased difference in AAD development. **A**, Rate of aortic rupture precipitated by the indicated groups in 2 and 4 weeks. ND: not detected; NS: not significant; non-ovx F: female mice with intact ovaries. **B**, Aortic growth over four weeks of challenge with BAPN+AngII. Data were analyzed using unpaired t-test. Please note that the differences of M vs. ovx+oil and F vs. ovx+E2 were not evaluated because the data were generated in different experiments.

Figure S4. Gross evaluation. Ovxed mice on E2 replacement or placebo were challenged with BAPN+AngII for four weeks. **A**, Aortas of the mice subjected to the indicated treatments. Segments below the level of diaphragm were cropped off. The gross pathology looked similar between the two groups. **B**, Uteri of the mice treated with E2 or placebo. Note that E2 replacement successfully prevented uteri from involution in ovxed mice.
